# Effectiveness of advice from physician and nurse on smoking cessation stage in Taiwanese male smokers attending a community-based integrated screening program

**DOI:** 10.1186/s12971-016-0080-0

**Published:** 2016-04-23

**Authors:** Dih-Ling Luh, Sam Li-Sheng Chen, Amy Ming-Fang Yen, Sherry Yueh-Hsia Chiu, Ching-Yuan Fann, Hsiu-Hsi Chen

**Affiliations:** Department of Public Health, Chung Shan Medical University, No.110, Sec. 1, Chien-Kuo N. Road, Taichung, 40201 Taiwan; Department of Family and Community Medicine, Chung Shan Medical University Hospital, Taichung, Taiwan, ROC; School of Oral Hygiene, College of Oral Medicine, Taipei Medical University, Taipei, Taiwan; Department of Health Care Management, College of Management, Chang Gung University, Taoyuan, Taiwan; Department of Health Industry Management, Kainan University, Taoyuan, Taiwan; Graduate Institute of Epidemiology and Preventive Medicine, College of Public Health, National Taiwan University, Room 533, No. 17, Xuzhou Road, Taipei, 10002 Taiwan

**Keywords:** Community, Intervention, Smoking cessation, Transtheoretical model

## Abstract

**Background:**

A screening program provides a teachable moment for primary prevention such as encouraging smoking cessation. However, little is known about the efficacy of smoking cessation intervention delivered to the general population through a community-based screening program.

**Methods:**

A quasi-experimental untreated control design with pre-test and post-test was conducted with 42 subjects receiving advice from physician and nurses (the PNA group), 39 receiving an informational leaflet (the leaflet group), and 308 control subjects.

**Results:**

The overall rate of reaching the action stage was 25 %, 5.7 %, and 7.8 in the PNA group, the leaflet group, and the control group, respectively. In approximately 45–60 % of all participants, the stage remained unchanged. Such an association between the intervention groups and stage changes was statistically significant (*p =* 0.02). The PNA group was more likely to have the improvement of stage (forward transition toward action stage) than the control group [adjusted odds ratio (aOR) = 2.27 (1.07–4.84)]. Deterioration (backward transition toward precontemplation) in the PNA intervention group was 37 % lower than that in the control group [aOR = 0.63 (0.20–2.01)].

**Conclusions:**

This study demonstrated that smoking cessation advice from physician and nurse is conducive to smoking cessation, as shown by greater movement toward and less movement away from smoking cessation through a community-based integrated screening platform.

**Electronic supplementary material:**

The online version of this article (doi:10.1186/s12971-016-0080-0) contains supplementary material, which is available to authorized users.

## Background

The efficacy of brief advice provided by health care professionals in smoking cessation program has been demonstrated to detect a significant increase in the rate of quitting by 66 % in comparison with no advice by polling data from 17 trials [1]. In spite of the finding from the trials, it would be more informative if a further study is conducted to throw light on why and how it works by assessing the dynamic changes of processes in relation to smoking cessation based on the transtheoretical model (TTM) [2]. Instead of treating smoking cessation as a dichotomous status (smoking and quitting), the TTM model considers smoking cessation a complex and continuous cyclic processes [3]. It describes and explains different stages of behavior change and the process of change by defining five specific stages: precontemplation, contemplation, preparation, action, and maintenance) [4–9].

In addition to the choice of theoretical model, setting for smoking cessation play an important role. The most common setting for delivery of smoking cessation advice was the primary care setting, followed by hospital wards, outpatient clinics, and industrial clinics [1]. Intervention has been rarely designed to be delivered in a community setting and targeted at the general population. With the advent of population-based screening for cancers and chronic diseases, it has been advised that screenings may provide an opportunity for teaching smoking cessation [10], including self-help information [11], physician referral for abnormal computed tomography findings [12], and smoking cessation advice from physicians [13]. For example, the smoking cessation programs have been jointly conducted with those screening programs for lung cancer [10–23] and also for cervical cancer [24, 25].

Following the model built in Keelung Community-based Integrated Screening program [26–29], Nantou County’s community-based integrated screening (CIS) (see below) aimed to deliver out-reaching screen service into communities for residents aged 20 years or over in Nantou. It is therefore possible for using the CIS as the platform for delivering a smoking cessation intervention for the underlying population.

The aim of this study was to evaluate the effects of two intervention strategies, advice on smoking cessation from physician and nurses and a self-contained informative leaflet, on the transitions through smoking-cessation stages (including forward transition toward the action stage and the backward transition toward precontemplation) through a community-based integrated screening program. Because the smoking prevalence rates were 46.9 % for males and 4.6 % females in Taiwan, respectively [30], we only included men subjects in the current analysis.

## Methods

### Study population

Subjects were selected from those who smoked and attended a community-based integrated screening program in Nantou, the central county in Taiwan, a multiple-screening model that has been described in full elsewhere [26–29]. In brief, the Health Bureau of Nantou County developed a program following the Keelung community-based integrated screening program (KCIS), which is tailored for early detection of multiple diseases including five cancers (breast cancer, cervical cancer, oral cancer, and colorectal cancers, and hepatocellular carcinoma) and three chronic diseases (type 2 diabetes, hyperlipidaemia, and hypertension). The recruitment criterion was in light of self-reported smoking status obtained from the questionnaire which was administrated by screening attendees themselves at on-site screening. Because there were few female smokers (2.6 % of smokers), study population were limited to males only.

All participants provided individual written informed consent during the uptake of screening asked their permission to link their screening data to external data such as national mortality for evaluating the survivorship of each participant for research purpose only. The current analysis had been approved by the local ethical committee of Nantou Health Bureau. All procedures met requirements mandated by the ethical guidelines for research in Taiwan today, including no-harms to participants, informed contents, privacy consideration, no interest conflict, prudent sample size calculation, and the freedom to withdraw from study. The research project was approved by local health authority to meet any ethical requirement mandated by Taiwanese government.

### Study design, intervention, and data-collection protocol

A quasi-experimental untreated control design with pre-test and post-test was adopted. The intervention was related to smoking cessation. Pre-test and post-testing were to measure the stage changes in smoking cessation following the transtheoretical model. The selection of intervention group and the control group is delineated as follows. Ten out of thirteen towns/villages in Nantou County were involved in the current smoking cessation study. Three were excluded due to the consideration of feasibility of local health manpower.

The smoking status was self-reported by a questionnaire. The question was ‘Do you have smoking habit currently?’ the answer included (1) never smoke (2) quitted and (3) smoke currently. Because the study population were current smokers, the quitter was define as those who had quit at follow-up telephone survey.

A total of 6,372 subjects (638 in intervention group and 5,734 in the control group) attended the Nantou Community-based Integrated Screening (NT-CIS) from February to September 2003. According to screening data, 103 smokers were identified in the intervention area out of 638 participants, and the smoking rate of the intervention group was 16.14 %. We provided screening results for participants within 2 weeks after on-site screening. The participants in the intervention area were classified according to whether they came back to consult with the screening report in person. Among the male smokers, 40 smokers took screening report at health station in person and received smoking cessation advice from physician and nurses directly. This group was defined as the ‘physician- and nurse-advised group’ (PNA group). Another 53 smokers who did not come back to take screening report in person, defined as ‘the leaflet group’, received smoking cessation advice, a smoking cessation informational leaflet, and their screening report by mail.

For intervention groups, PNA group received advice from physician including (1) a brief summary on smoking participant’s age, smoking history, and chronic disease history accrued from screening questionnaire, (2) biochemical examination results from on-site screening, and (3) a formal documentation of audience on smoking cessation signed by physician. The average time to persuade participants to stop smoking through physician was 42 s. Participants who were introduced by the advice of smoking cessation upon recommendation by physician signature were taken by public health nurse to assess the willingness of stop smoking and the degree of addiction to smoking, and provide relevant information on smoking cessation for them. Those who failed to take the results of first stage of screening were noticed by mail with recommendation on smoking cessation and relevant information, harm of smoking, and benefit of smoking cessation on health together with the results of the first stage of screening.

In the control group, there were 5,734 participants and 1,065 smokers. The smoking rate of the control group was 18.57 %. We chose 390 male smokers (approximately four times the number of study subjects in the intervention groups) from the control group by simple random sampling. This group, defined as ‘the control group’, was still invited to undertake the routine screening but none of any formal smoking cessation advice either from physician or nurse directly or from the mailed leaflet was provided.

**Fig. 1:**
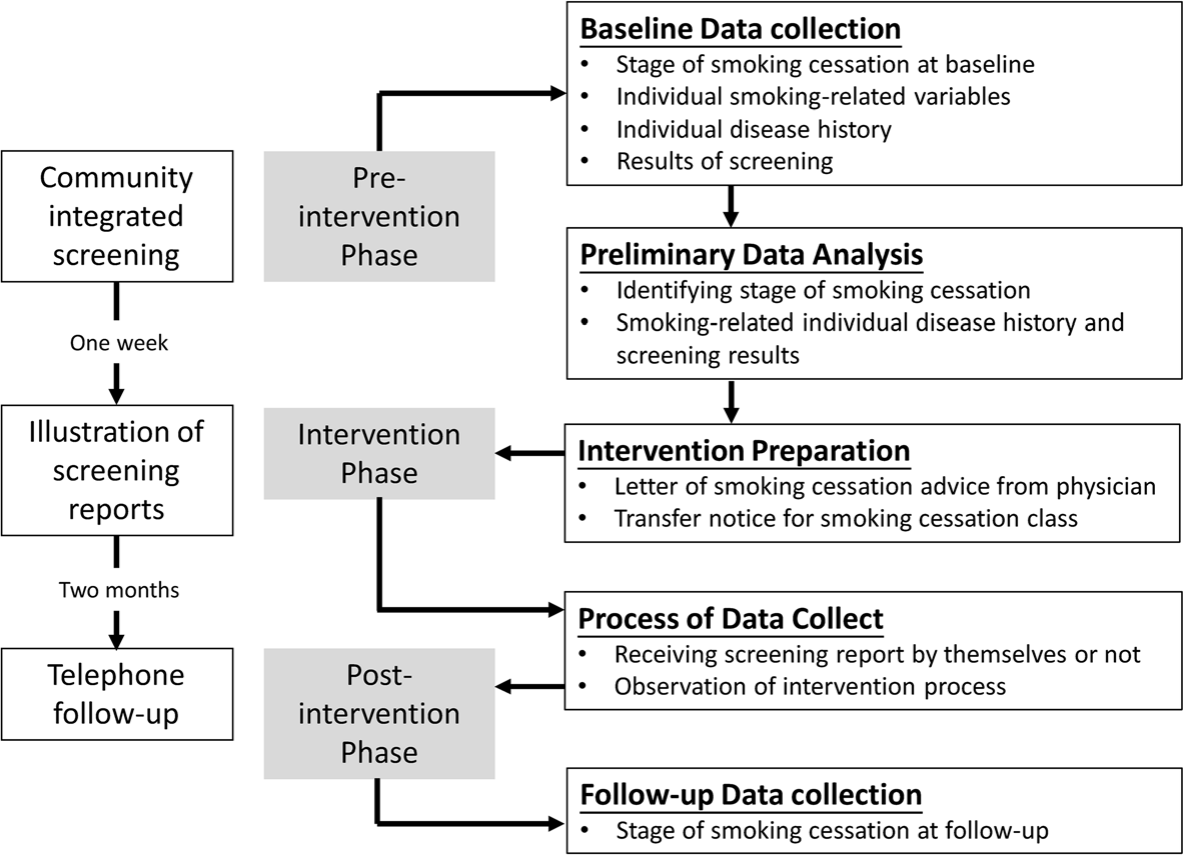
Implementation procedure and data collection in 2003

**Fig. 2:**
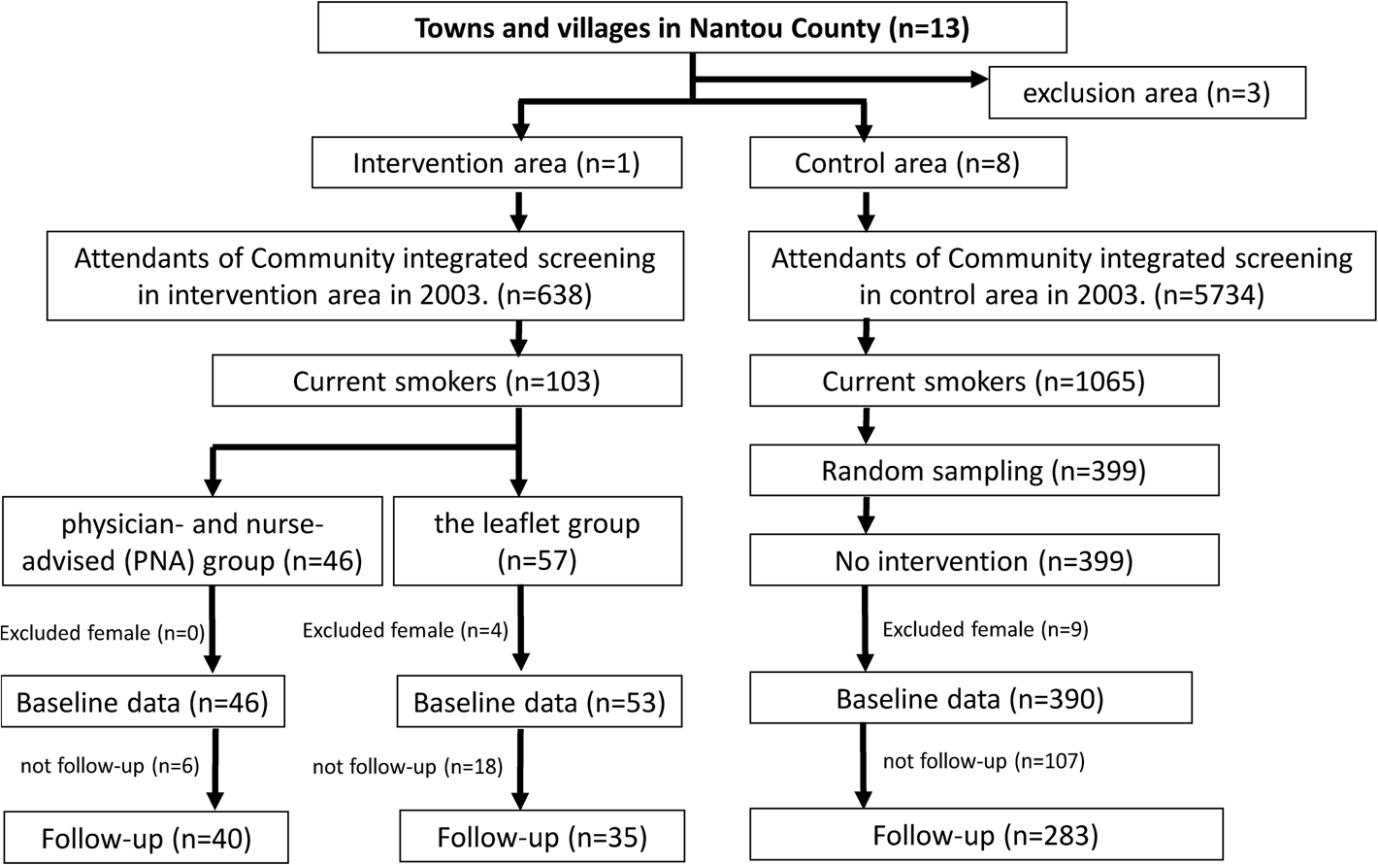
Participant flow in quasi-experimental untreated control design with pre-test and post-test effectiveness of advice from physical and nurse on smoking cessation stage

Public health nurses followed all three groups (namely PNA, leaflet, and control groups) by telephone from October to December 2003, approximately 2 to 8 months after screening, to interview them in order to glean information on smoking cessation stage. Figure 1 summarises the procedure for implementing the current study and collecting data. The details of procedure are given in Additional file 1. The participant flow chart is shown in Fig. 2.

According to our sample size, the statistical power using multivariable logistic regression model was approximately 64.3 % for PNA group versus the control group, and 54.3 % for the comparison of three groups when the leaflet group (with the smallest sample size) were added.

### Instrument and definition of variables

Data sources for this study included screening data and follow-up telephone survey data. Baseline variables from the questionnaire included age (≤50, 51–64 or ≥65 years), smoking commencement age (<20, ≥20 years), time of first cigarette in the morning (<30 or ≥30 min after waking), smoking cessation advice from others in the previous 6 months (yes/no), and personal disease history (hypertension, diabetes).

The primary outcome variable was the change in smoking cessation stage, as measured by the response to two questions: ‘are you a current smoker?’ and ‘have you ever seriously considered stopping smoking, and if so, when do you plan to stop?’ Possible responses to the first question were ‘yes’ and ‘no’. Responses to the second question included ‘never’, ‘yes, but not sure when I will quit’, ‘yes, and I intend to quit in the next 6 months’, ‘yes, and I intend to quit within one month’, and ‘I have quit smoking’ (this response was in the follow-up questionnaire only). Following the transtheoretical model [6, 31, 32], the smoking cessation stages at baseline were divided into three categories: precontemplation (smokers who have never considered quitting), contemplation (smokers who have considered quitting), preparation (smokers who have considered quitting and intended to quit in the next 6 months and this month). The smoking-cessation stage at follow-up was categorized into one of four stages, precontemplation, contemplation, preparation, and action (smokers who had quit at follow-up).

The outcome variable was the change in smoking cessation stage, which was classified in one of three categories at follow-up based on a comparison with the stage at baseline: ‘improvement’, ‘deterioration’, and ‘no change’. Improvements included the change from precontemplation at baseline to contemplation, preparation, or action at follow-up; the change from contemplation at baseline to preparation or action at follow-up; and the change from preparation at baseline to action at follow-up. Deterioration included the change from contemplation at baseline to precontemplation at follow-up and the change from preparation at baseline to precontemplation or contemplation at follow-up. ‘No change’ referred to smokers whose stage at follow-up was the same as that at baseline.

### Statistical analysis

Descriptive statistics (frequency and percentage) were used to describe the distribution of demographic and smoking-related control variables. We used chi-square and *t*-tests to examine differences between baseline information and intervention. The Fisher’s exact test was used to examine differences between categories when cell numbers are small. To assess the effects of intervention on the changes in smoking-cessation stages given the low number of participants, we defined three categories of stage change: improvement, deterioration, and no change, as mentioned above. We used frequency and percentage to show distribution of smoking cessation stage at follow-up survey by stage at baseline and three groups. Then, we used the chi-square test to examine the relationships among the three categories of smoking cessation stage change and related factors, which included age, smoking commencement age, time of first cigarette in the morning after waking, cessation advice from others, and intervention group. Multinomial logistic regression was conducted to estimate the intervention effect after adjustment for other significant variables. A significance level of 5 % was set for statistical significance. All analyses were conducted using SAS version 9.2.

## Results

### Descriptive results at baseline

Table 1 shows the frequencies of sociodemographic characteristics, smoking-related behaviour, and history of chronic disease at baseline among male smokers. There were lacking of difference across three groups, PNA, leaflet, and control group with respect to smoking-related behaviours, as shown in Table 1. There were also lacking of statistically significant differences across the three groups with respect to diabetes, and hypertension.

**Table 1:** Distribution of socio-demographic, smoking related attributes, chronic disease, and biochemical markers among male smokersat baseline. (*N* = 489)

variables	Total	%	Intervention						*χ* ^2^ (degree of freedom
			P&NA Group		Leaflet Group		Control		
			*n*	%	*n*	%	*n*	%	*p* value
	489		46	9.4	53	10.8	390	79.8	
Age Group									
≦50	96	19.6	3	6.5	10	18.9	83	21.3	*χ* ^2^_(df=4)_ = 8.27
51–64	119	24.3	9	19.6	12	22.6	98	25.1	*p* = 0.08
≧65	274	56.0	34	73.9	31	58.5	209	53.6	
Smoking related attributes									
Smoking commence age									
<20	121	26.0	14	33.3	12	23.1	95	25.7	*χ* ^2^_(df=4)_ = 3.52
20	156	33.5	15	35.7	14	26.9	127	34.3	*p* = 0.48
>20	188	40.4	14	33.3	26	50.0	148	40.0	
NK	24		3		1		20		
Time for first cigarette at morning									
<30 min	286	60.7	29	64.4	34	68.0	223	59.3	*χ* ^2^_(df=2)_ = 1.69
≧30 min	185	39.3	16	35.6	16	32.0	153	40.7	*p* = 0.43
NK	18		1		3		14		
Smoking cessation advice from others									
yes	167	36.3	18	40.0	16	32.0	133	36.4	*χ* ^2^_(df=2)_ = 0.67
no	293	63.7	27	60.0	34	68.0	232	63.6	*p* = 0.72
NK	29		1		3		25		
Smoking cessation stage at baseline									
Precontemplation (PC)	285	61.0	33	73.3	34	66.7	218	58.8	*χ* ^2^_(df=4)_ = 5.96
Contemplation (C)	156	33.4	9	20.0	16	31.4	131	35.3	*p* = 0.20
Preparation (P)	26	5.6	3	6.7	1	2.0	22	5.9	
NK	22		1		2		19		
Chronic disease at baseline									
Diabetes history	40	9.6	3	3.5	4	7.7	33	10.0	*χ* ^2^_(df=2)_ = 0.28(*p* = 0.87)
Hypertension history	67	15.8	9	10.6	3	5.8	55	16.4	*χ* ^2^_(df=2)_ = 4.39(*p* = 0.11)

precontemplation (PC): smokers who never consider to quit

contemplation (C): smokers who ever consider and intent to quit in the six months

preparation (P): smokers who ever consider and intent to quit in this month

*NK* not know

*PNA* Physician and nurse advice group

### Changes in smoking cessation stage

As shown in Table 2, smoking cessation stages at follow-up were significantly associated with those at baseline. Additionally, the changes in smoking cessation stage revealed that a higher percent of those who were at the preparation stage at baseline changed to the action stage in the follow-up survey (30 %) compared with those who were at the precontemplation (7.4 %) and contemplation (9.8 %) stages at baseline. The proportion of those in the action stage at follow-up was higher in the PNA group (25 %) than in the leaflet group (5.7 %) and the control group (7.8 %).

**Table 2:**
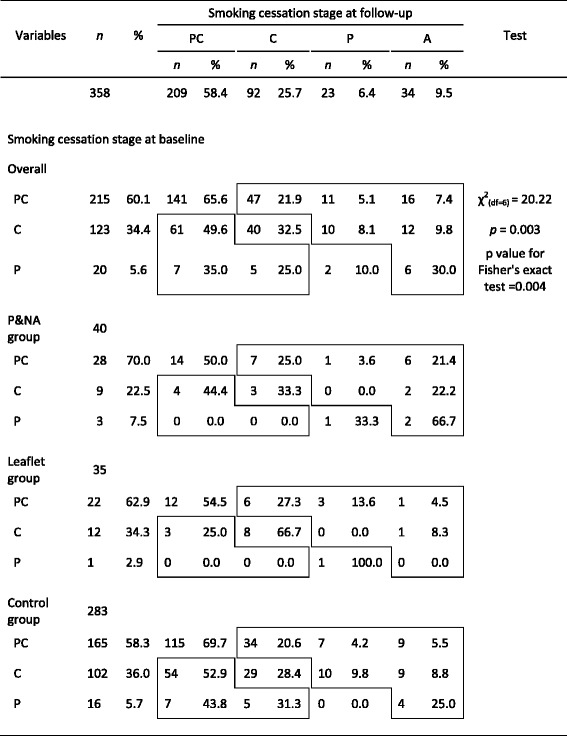
Distribution of smoking cessation stage at follow-up survey by stage at baseline and intervention group (*n* = 358)

Precontemplation (PC): smokers who never consider to quit

Contemplation (C): smokers who ever consider and intent to quit in the six months

Preparation (P): smokers who ever consider and intent to quit in this month

*NK* not know

The relationship between the intervention and smoking cessation stages before and after intervention were examined. As shown in Table 2, of those who were in the precontemplation category at baseline, the proportion who took action was higher in the PNA group (21.4 %) than in the leaflet (4.5 %) and the control groups (5.5 %). Of those who were in preparation stage at baseline, the proportion who took action was higher in the PNA group (66.7 %) than in the control (25.0 %) and leaflet groups (0.0 %). The absolute differences between the PNA group and the control group in the percentage of participants who moved from precontemplation, contemplation, and preparation to action were 15.9, 13.4, and 41.7 %, respectively.

The distribution of change as defined by three categories, improvement (*n* = 102), deterioration (*n* = 73), and no change (*n* = 183) with respect to age, smoking related factors at baseline, and intervention group, are shown in Table 3. The results revealed that 28.5 % of male smokers showed improvement in smoking stage, whereas 20.4 % deteriorated. The changes in stage were significantly associated with having received cessation advice from others (*p* = 0.04) but were not statistically associated with age of smoking commencement (*p* = 0.81) and time of the first cigarette upon waking in the morning (*p* = 0.45). The rate of improvement was considerably higher in the PNA group than in the control group (45 % versus 25.8 %), whereas the rate of deterioration was lower in the PNA group (10 % versus 23.3 %). A similar but less remarkable finding was noted for the leaflet group (see Table 3). Approximately 45–60 % of all participants were in the category of no change. The association between intervention group and the changes in stage was statistically significant (*p* = 0.02).

**Table 3:** Relationships between changes in smoking cessation stage and related factors (*n* = 358)

Variables	*n*	%	Change of smoking cessation stage						Chi-Square test	Crude OR(95 % C.I.)	
			No change^a^		Deterioration^b^		Improvement^c^			Improvement / No Change	Deterioration / No Change
			*n*	%	*n*	%	*n*	%			
	358		183	51.1	73	20.4	102	28.5			
Age group											
≦50	65	18.2	31	47.7	16	24.6	18	27.7	*χ* ^2^ = 1.58	1.00	1.00
51–64	90	25.1	46	51.1	20	22.2	24	26.7	*p* = 0.81	0.83 (0.37–1.88)	0.67 (0.28–1.58)
≧65	203	56.7	106	52.2	37	18.2	60	29.6		1.03 (0.51–2.07)	0.65 (0.31–1.36)
Smoking-related factors											
Smoking commence age											
<20	92	26.6	49	53.3	19	20.7	24	26.1	*χ* ^2^ = 1.59	1.00	1.00
20	121	35.0	62	51.2	21	17.4	38	31.4	*p* = 0.81	1.23 (0.64–2.36)	0.72 (0.34–1.55)
>20	133	38.4	67	50.4	30	22.6	36	27.1		1.10 (0.57–2.11)	1.05 (0.52–2.12)
NK	12		5		3		4				
First cigarette in the morning											
<30 min	210	60.9	102	48.6	43	20.5	65	31.0	*χ* ^2^ = 1.59	1.00	1.00
≧30 min	135	39.1	74	54.8	27	20.0	34	25.2	*p* = 0.45	0.71 (0.42–1.20)	0.80 (0.44–1.46)
NK	13		7		3		3				
Cessation advice from others											
no	121	35.6	69	57.0	15	12.4	37	30.6	*χ* ^2^ = 6.49	1.00	1.00
yes	219	64.4	105	47.9	52	23.7	62	28.3	*p* = 0.04	1.06 (0.63–1.79)	2.19 (1.13–4.22)
NK	18		9		6						
Intervention group											
Control	283	79.1	144	50.9	66	23.3	73	25.8	*χ* ^2^ = 11.20	1.00	1.00
PNA	40	11.2	18	45.0	4	10.0	18	45.0	*p* = 0.02	2.27 (1.07–4.83)	0.62 (0.20–1.95)
Leaflet	35	9.8	21	60.0	3	8.6	11	31.4		1.00 (0.44–2.26)	0.35 (0.10–1.22)

Precontemplation (PC): smokers who never consider to quit

Contemplation (C): smokers who ever consider and intent to quit in the six months

Preparation (P): smokers who ever consider and intent to quit in this month

Action (A): smokers who quit at follow-up

*PNA group* Physician and nurse advice group

^a^No change’ means that the stage at follow-up was the same as that at baseline

^b^Improvement included: 1.from precontemplation at baseline to contemplation, preparation, and action at follow-up; 2. from contemplation to preparation and action; 3. from preparation to action

^c^Deteriorate included: 1. from contemplation to Precontemplation; 2. from preparation to precontemplation and contemplation

Table 4 shows after adjusting for smoking cessation advice from others, individuals in the PNA group were more likely to show improvement than were those in the control group [adjusted odds ratio (OR) = 2.27 (95 % CI: 1.07–4.84)], whereas the improvement in the leaflet group was close to that in the control group [adjusted OR =0.99 (95 % CI: 0.44–2.25)].

**Table 4:** Relationship between change in smoking cessation stage and intervention using multinominal logistic regression (*n* = 358)

Variables	Change of smoking cessation stage			
	Improvement^a^ / No change^c^		Deterioration^b^ / No change^c^	
	OR	95 % CI	OR	95 % CI
Cessation advice from others				
No	1		1	
Yes	1.05	0.62–1.78	2.21	1.14–4.29
Intervention group				
Control	1		1	
PNA	2.27	1.07–4.84	0.63	0.20–2.01
Leaflet	0.99	0.44–2.25	0.33	0.09–1.15

*PNA* Physician and nurse advice group

Independent variables included cessation advice from others and intervention group in the multinominal logistic regression

^a^Improvement included: 1.from precontemplation at baseline to contemplation, preparation, and action at follow-up; 2. from contemplation to preparation and action; 3. from preparation to action

^b^Deterioration included: 1. from contemplation to Precontemplation; 2. from preparation to precontemplation and contemplation

^c^‘No change’ means that the stage at follow-up was the same as that at baseline

The deterioration in the PNA group was 37 % lower than that in the control group [adjusted OR = 0.63 (95 % CI: 0.20–2.01)] (Table 4). The deterioration in the leaflet group was 67 % lower [adjusted OR = 0.33 (95 % CI: 0.09–1.15)] than that in the control group (Table 4). When the results for the two intervention groups were combined, the decrease in deterioration was 54 %, although the results failed to reach statistical significance [adjusted OR = 0.46 (0.19–1.09)] (data not shown). It is interesting to note that those who had received cessation advice from others were more likely to show deterioration than were those who had not received such advice [adjusted OR = 2.21 (95 % CI: 1.14–4.29)]; however, the influence on improvement was not statistically significant [adjusted OR = 1.05 (95 % CI: 0.62–1.78)].

## Discussion

Applying the TTM model to smoking cessation program implemented through an integrate screening platform, we found the advice from physician and nurses (the PNA group) could significantly enhance change toward the action stage (forward transition) for quitting smoking and reduce (albeit not statistically significantly) the possibility of regressing to a stage farther from the action stage (backward transition). The strategy provided with only leaflet (the leaflet group) for participants could not have such benefit. Of the smoking-related factors, taking advice from others was conducive to regression to a stage farther from the action stage, whereas its effect on movement toward the action stage was small. Other smoking-related factors such as age of smoking commencement and time of first cigarette after waking in the morning did not statistically influence improvement or deterioration in smoking cessation stage.

### Effects of physician and nurse advice on the change in smoking cessation stage

Our major intervention program was based on advice from physician and nurses, as this is often regarded as a potentially efficient approach [1]. Our main finding was consistent with the results from systematic review that concluded that physician advice and a good booster from nurse’s consultation and advice for smokers to quit will increase the rate of smoking cessation [1, 33–35].

However, as seen in previous studies, most physicians have not routinely asked about their patients’ smoking status [36, 37], and most smokers did not receive advice to quit from their physician [38, 39]. Furthermore, a large proportion of primary care physicians did not follow recommendations to promote smoking cessation among their patients [40]. The barriers to offering smoking prevention counselling included lack of training in smoking cessation [37], lack of patient educational materials, and lack of time [41]. On average, smokers were less likely to have received advice to quit if they were single (compared with divorced, widowed, or separated), had higher levels of education, were lighter smokers, had no previous quit attempts, and had physicians who smoked [36]. It is timely to provide an easier and quicker reminder method for physician to give smoking cessation on smoking clients during screening process. Our study designed a simple smoking cessation advice form as a reminder to the physician.

### Effects of informative leaflet distribution on smoking cessation stage

We did not see a benefit of the intervention that entailed distributing the self-contained informational leaflet in our study, which was consistent with result from a previous study [13]. Among current smokers who underwent low-dose fast spiral chest CT for lung cancer screening, those who received standard written self-help materials or a written list of internet resources for smoking cessation showed no statistically significant change in the 7-day point prevalence quit rates or advancement in motivational readiness to stop smoking compared with the control [13].

### The cyclic smoking cessation process using a transtheoretical model

The proposed analysis method for our data is unique because we focused not only on stage changes from precontemplation to action but also on changes in the reverse direction, e.g., from preparation to precontemplation. Shedding light on these cyclic processes is informative for understanding the factors that may account for stage changes indicating improvement, i.e., movement toward action, and deterioration, i.e., movement away from action. Identifying both promoters and inhibitors of smoking cessation gives clues for designing an effective intervention program. Analysis of these cyclic processes has not been considered in previous screening-based intervention studies addressing changes in smoking cessation stage [16, 22].

In our finding, in addition to forward transition, the deterioration rates in the control group, i.e., movement from preparation to contemplation or to precontemplation, were considerably higher than those in the PNA group (75.1 % versus 0 %). The deterioration rate from contemplation to precontemplation was only slightly higher in the control than in the PNA group (52.9 % versus 44.4 %), suggesting that smokers in the contemplation stage at baseline had the same chance of regressing to precontemplation regardless of the intervention group or the control group.

Our study found that those who had received cessation advice from others were more likely to show deterioration than were those who had not received such advice. Few studies have been conducted to address why advice from others showed an adverse effect for smoking cessation. It is possible that advice from others in our study may contain information that decrease the negative outcome expectations of smoking and the positive outcome expectations of quitting. It requires a further study to clarify this cause.

### Limitations and suggestions

There are pros and cons of the current study. Strength of the current study are that the intervention program in a community setting is relatively inexpensive and accessible to community residents. The weaknesses resulting from the expediency of this community-based study is a lacking of randomized controlled study design and is limited to the allocation of participants to two interventions using other areas without intervention program as the control group. However, as we have considered several confounding factors, particularly smoking behaviours the results are supposed to be credible and comparable to that if the randomized controlled trial design is adopted. As participants were not randomly assigned to the intervention groups (PNA and informational leaflet) the group-assignment of participants in our study to the PNA group or the leaflet group was highly affected by depended on whether the participants came back in person to receive the first-stage screening report. Such an assignment may introduce bias, e.g. participants who returned to take the screening in person and were assigned to the PNA group may be more motivated to stop smoking compared to participants who did not return and were assigned to the leaflet group. However, we believe this concern may not be serious due to the fact that there were lacking of statistical significance differences across intervention groups with respect to baseline characteristics. Admittedly, we have no information about the maintenance of stage changes. The small sample size of some groups may limit the results, particularly in regard to deterioration in stage changes. As far as statistical power is concerned, although the optimal ratio of case to control group in term of cost and effect- size related to statistical power is 1 to 4, the ratio of case to the control group increased to 1 to 9 in the current study may increase cost but may not mitigate statistical power.

## Conclusion

A community-based smoking cessation intervention with a transtheoretical underpinning demonstrated that advice on smoking cessation from physicians and nurses is effective in smoking cessation, as evidenced by improvement in the stage of smoking cessation and a reduction in regression toward precontemplation.

## Additional file

Additional file 1: Implementation procedure and data collection. (DOC 25 kb)

## Abbreviations

CIS: Community-based integrated screening
OR: odds ratio
PNA: physician- and nurse-advised group
TTM: Transtheoretical model
