# Supplementary material for: Effectiveness of advice from physician and nurse on smoking cessation stage in Taiwanese male smokers attending a community-based integrated screening program
Source: Tob Induc Dis. 2016 Apr 23;14:15. doi: 10.1186/s12971-016-0080-0 (PMC4841961; doi:10.1186/s12971-016-0080-0)
Supplement: Additional file 1: — Implementation procedure and data collection. (DOC 25 kb) [file 12971_2016_80_MOESM1_ESM.doc]

**Appendix**

**Implementation procedure and data collection**

Before intervention, we collected baseline data including demographic characteristics, life-style factors, results of screening, stage of smoking cessation, and smoking-related variables. Subjects who declared themselves current smokers were eligible for the smoking-cessation program, which included advice from health professionals and the screening report. Information provided for physicians` participating in the smoking cessation program included age, sex, smoking status, health-related behaviour (drinking and betel chewing), personal disease history, and the physician’s sworn signature given to the smokers who were advised to stop smoking. This was called the pre-intervention phase.

When a smoker was ascertained from smoking cessation advice in screening report, physician participated in the intervention phase by giving formal smoking cessation advice with a statement signed in front of the smoker. In addition to providing the smoking cessation self-help pamphlet and other related information, the implementation of smoking cessation education, which was conducted by public health nurses (PHNs), varied with the smoker’s cessation stage. Smoking participants who did not come back to receive their screening reports in person received the smoking cessation advice with the physician’s signature, the smoking cessation leaflet, and their screening report by mail.

Due to the restriction of study period, a follow-up telephone survey was conducted 2 months after the delivery of the screening reports to participants. In the intervention area, 81 of 103 participants completed the follow-up survey, a 78.6% of response rate. Of these 81 participants, 42 were in the PNA group (completion rate of 91.3%), and 39 were in the leaflet group (completion rate of 68.4%). In the control group, 308 of 399 participants completed the follow-up survey (completion rate of 77.2%). There was no significant difference between the intervention group and the control group with respect to completion rate (χ^2^_df=1_ = 0.0984, *p =* 0.7538). Among the intervention group, the completion rate of the PNA group was higher than that of leaflet group (chi-square = 7.94, *p =* 0.0048).

Among 502 participants, 13 females (9 in intervention and 4 in control group) were excluded as sample sizes were too small for analysis. Thus, 489 participants were available for analysis with baseline characteristics (sociodemographic, smoking related behaviors, and chronic diseases among male smokers) shown in Table 1. Of the 489 male participants, 22 did not identify their smoking cessation stage at baseline. Of the 467 participants who were interviewed at baseline, 109 (23.34%) did not provide their smoking cessation stage in the follow-up survey.

There was lacking of statistical difference between respondents and non-respondents in the follow-up survey with respect to baseline characteristics. Therefore, 358 participants with complete data were included in the following analysis.
